# Religion and Completed Suicide: a Meta-Analysis

**DOI:** 10.1371/journal.pone.0131715

**Published:** 2015-06-25

**Authors:** Andrew Wu, Jing-Yu Wang, Cun-Xian Jia

**Affiliations:** 1 School of Medicine, Vanderbilt University, Nashville, United States of America; 2 Department of Epidemiology, School of Public Health, Shandong University, Jinan, China; 3 Center for Suicide Prevention Research, Shandong University, Jinan, China; Catholic University of Sacred Heart of Rome, ITALY

## Abstract

**Introduction:**

Suicide is a major public health concern and a leading cause of death around the world. How religion influences the risk of completed suicide in different settings across the world requires clarification in order to best inform suicide prevention strategies.

**Methods:**

A meta-analysis using search results from Pubmed and Web of Science databases was conducted following PRISMA protocol and using the keywords “religion” or “religious” or “religiosity” or “spiritual” or “spirituality” plus “suicide” or “suicidality” or “suicide attempt”. Random and fixed effects models were used to generate pooled ORs and I^2^ values. Sub-analyses were conducted among the following categories: young age (<45yo), older age (≥45yo), western culture, eastern culture, and religious homogeneity.

**Results:**

Nine studies that altogether evaluated 2339 suicide cases and 5252 comparison participants met all selection criteria and were included in the meta-analysis. The meta-analysis suggested an overall protective effect of religiosity from completed suicide with a pooled OR of 0.38 (95% CI: 0.21–0.71) and I^2^ of 91%. Sub-analyses similarly revealed significant protective effects for studies performed in western cultures (OR = 0.29, 95% CI: 0.18–0.46), areas with religious homogeneity (OR = 0.18, 95% CI: 0.13–0.26), and among older populations (OR = 0.42, 95% CI: 0.21–0.84). High heterogeneity of our meta-analysis was attributed to three studies in which the methods varied from the other six.

**Conclusion:**

Religion plays a protective role against suicide in a majority of settings where suicide research is conducted. However, this effect varies based on the cultural and religious context. Therefore, public health professionals need to strongly consider the current social and religious atmosphere of a given population when designing suicide prevention strategies.

## Introduction

Suicide is a leading cause of death worldwide. It is responsible for over 800,000 deaths per year and ranks among the top three leading causes of death for 15–44 year olds [1–3]. In fact, as a response to this ongoing global burden, the World Health Organization (WHO) recently called for increased prioritization of suicide prevention in a 2014 report [3, 4]. In order to inform efforts to tackle this tragic, yet preventable issue, many studies with most conducted in the U.S. and Europe have evaluated a variety of possible contributing factors [5, 6]. However, whether the relationships between some of these evaluated factors and suicide is definitively positive or negative among all settings remains undecided.

One such factor is religion, which has long been an established factor in the overall health of an individual [5, 7], whether the working mechanism is through psychological stability, empowerment, social networking, particular religious rituals, lifestyle patterns, or other undiscovered factors [5]. It may be unsurprising then that according to numerous large polls, religion plays an important role in a majority of people’s lives [8, 9]. For example, a 2010 Gallup poll of 114 countries revealed the median proportion of adults reporting religion to be an important part of their daily lives was 84% [10]. Another more recent poll in 2012 by Gallup International reported only 23% of 50,000 participants across 57 countries claiming to be non-religious [11].

The existence of a relationship specifically between suicide and religion has been well-documented in numerous reviews [5, 7, 12–15]. Most studies reveal that increased religiosity protects against suicide [5, 16–22], but other studies conducted in unique settings have also suggested an association with increased suicide [23, 24]. Additionally, suicide literature seems to comprise of more studies focusing on suicide attempts than those studying completed suicide as an outcome [25–31], and many other studies use methods that are subject to ecological fallacy [6, 32–36]. Given the global prevalence of religious beliefs, the severe burden of suicide, and the relative dearth of literature on completed suicide, it becomes apparent to clarify the relationship between completed suicide and religion.

Taken together, in light of recent evidence that suggests a positive relationship between religion and suicide as well as the urgent need to implement suicide prevention strategies, this meta-analysis attempts to clarify the relationship between religion and completed suicides among a variety of populations.

## Materials and Methods

### Search Strategy

This study was conducted according to PRISMA guidelines (http://www.prisma-statement.org/). Searches were conducted using the Pubmed and Web of Science databases. Investigators used the key words “religion” or “religious” or “religiosity” or “spiritual” or “spirituality” plus “suicide” or “suicidality” or “suicide attempt” to search for articles. The last term was included since studies that assess completed suicides may also include suicide attempts. Two investigators (AW and JW) independently conducted the literature search on March 16^th^, 2015 according to the strategy above. Disagreements were resolved by discussion. Additional articles were also solicited from an expert in the field.

### Eligibility Criteria

An initial screen was based on the following criteria: 1) studies used religion as an exposure and completed suicide as an outcome; 2) article was written in English; 3) literature published during or between 2000 and 2015; 4) no overlap of subjects or data from the same study population; 5) only used human subjects; and 6) provided comparison data on religiosity among study subjects. Regarding this last criterion, odds ratios (OR), rate ratios, and relative risks were included. Studies that did not provide odds ratios were still included if they could be calculated with 2 x 2 cross tables. Therefore, narratives and qualitative studies were excluded. Only literature published between 2000 and 2015 were included in order to capture the most recent trends in religion. This is in regard to the enormous changes religion tends to undergo throughout history–specifically in how it affects worldviews and culture [5, 7, 37].

### Data Extraction

Information extracted from the study included the title, name of the first author, year of publication, journal name, country of study population, study design, qualitative description of the control or comparison population, and how religion was measured. For the meta-analysis, we specifically extracted the total number of cases, total number of controls, the number of cases labeled as “religious” and those labeled as “non-religious”, the number of comparison participants or controls labeled as such, and the adjusted odds ratios (OR’s) with their respective 95% confidence intervals (CI) if provided. In order to analyze sub-groups, we also obtained the average ages and which studies were conducted in areas that were religiously homogenous (where primarily one religion was dominant) which was determined per the content of the article.

### Literature Quality Assessment

The Newcastle-Ottawa Scale (NOS) for Assessing the Quality of Nonrandomized Studies in Meta-Analysis was utilized to quantitatively evaluate the quality of the studies [38]. All studies were scored individually according to each item and then totaled.

### Statistical Analysis

All pooled results and 95% CI’s were log transformed and used to obtain the pooled OR and 95% CI. If the adjusted OR’s and CI’s were unavailable, unadjusted OR’s and CI’s were calculated using the data provided in the study article. Meta-analyses were conducted for unadjusted and adjusted risk estimates separately as a sensitivity analysis. If the heterogeneity among the studies was large (*I* ^2^>50%), a random effects model (REM) was used to calculate the pooled OR, and sequential omission of studies was conducted using an algorithm (‘hetred’ command in Stata) to determine which studies contributed the most heterogeneity. Otherwise (*I* ^2^>50%), a fixed effects model (FEM) was applied. The pooled results are presented on the exponential scale. Publication bias was evaluated with the Begg adjusted correlation rank test and Egger regression asymmetry test. Sub-groups were created with the following categories: young age (if study population mean age < 45yo), eastern or western-based cultures, and studies where the setting demonstrated religious homogeneity. Meta-regressions were performed to explore the overall moderating effect in each sub-analysis and on the variable of study design. An influence analysis was also conducted to validate the stability of the data via sequential removal of each study. All analyses were performed in Stata 12.0 and statistical significance was indicated at p = 0.05.

## Results

### Study Characteristics

**Fig 1:**
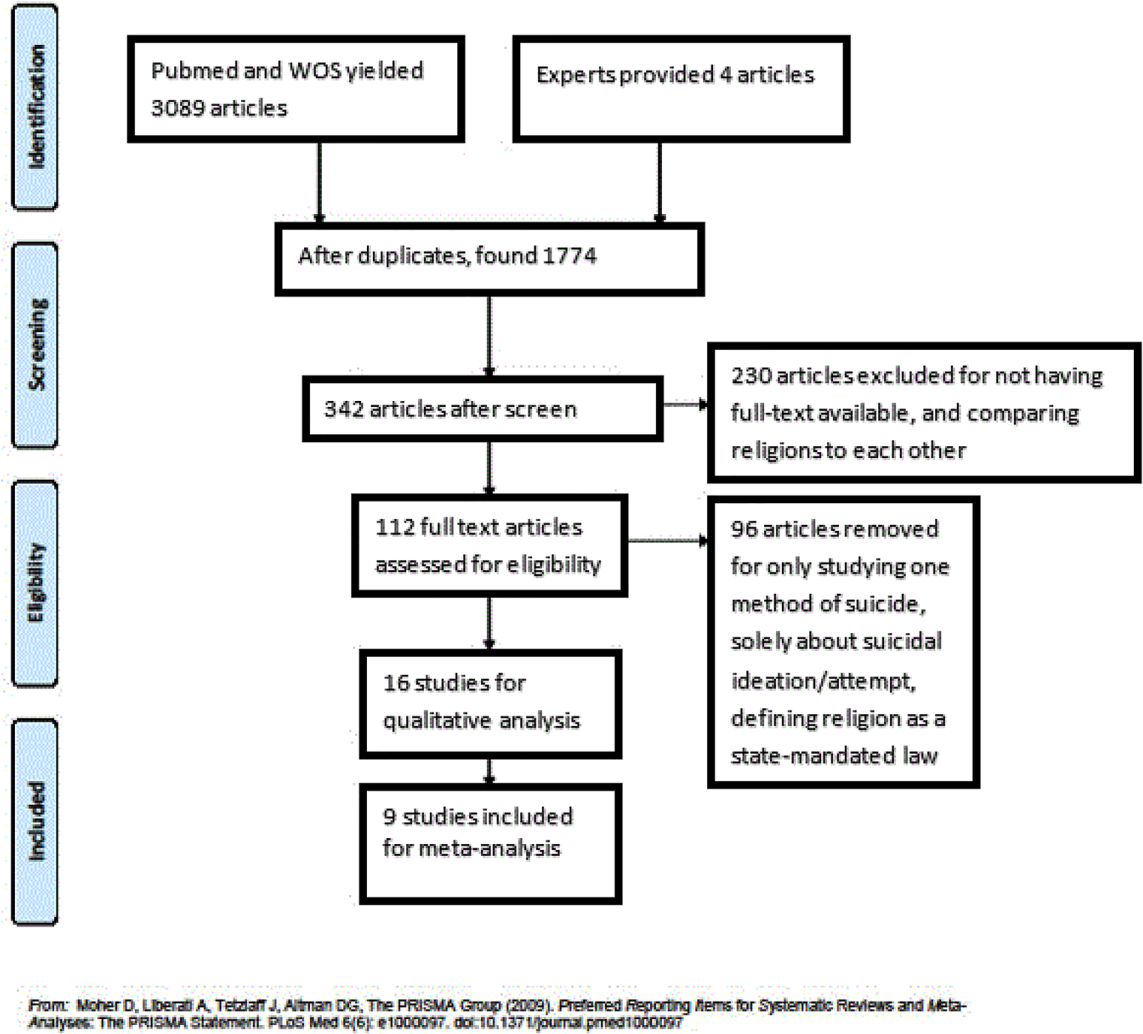
PRISMA 2009 Flow Diagram. PRISMA flow diagram with reasons for filtering articles.

Searches on Pubmed and Web of Science using the key word searches aforementioned generated 3089 articles, and 4 additional articles were provided by an expert in the field. After eliminating duplicates, 1,774 articles remained. The PRISMA flow diagram for this process can be seen in Fig 1. Ultimately, we found nine potential articles after removing studies according to our criteria.

Seven studies were case-control studies (six of which used psychological autopsy methods) and two were retrospective cohort studies. Altogether, these studies included 2339 completed suicides and 5252 individuals who were either living comparisons or comparisons who had died natural deaths. Detailed descriptions of our studies are found in Table 1. Using the NOS method to evaluate the quality of these studies, one study had 8 stars [24], four studies had 7 stars [16–19], one study had 6 stars [21], and three studies had 5 stars [20, 22, 23]. Of note, none of the psychological autopsy case-control studies were able to satisfy the criteria for “ascertainment of exposure” because the exposure of religion was assessed through interviews that could not been blinded since the desired outcome was completed suicides.

**Table 1:** Descriptive information of literature included in meta-analysis.

First Author	Year	Location	Study Design	Total Cases	Total Controls	Definition of “Religious”
Fellingham	2000	USA	Retrospective cohort	59	156	Active church participation
Nisbet	2000	USA	Case-control	584	4279	Church attendance
Hilton	2002	USA	Retrospective cohort	95	222	Active church participation
Duberstein	2004	USA	Case-control psychological autopsy	86	86	Practicing a religion
Zhang	2004	China	Case-control psychological autopsy	66	66	Claiming a religion
Tsoh	2005	China (Honk Kong)	Case-control psychological autopsy	67	91	Religion considered salient
Almasi	2009	Hungary	Case-control psychological autopsy	192	192	Practicing a religion
Kurihara	2009	Indonesia (Bali)	Case-control psychological autopsy	60	120	Practicing a religion (temple anniversary attendance)
Zhang	2010	China	Case-control psychological autopsy	392	416	Claiming a religion

Articles are arranged in order of publication year.

### Meta-analysis and Sub-analyses

We analyzed all 9 studies with a random effects analysis that revealed a pooled OR of 0.38 (95% CI: 0.21–0.71) and I^2^ of 91%, suggesting a protective effect of religion against completed suicide.

**Fig 2:**
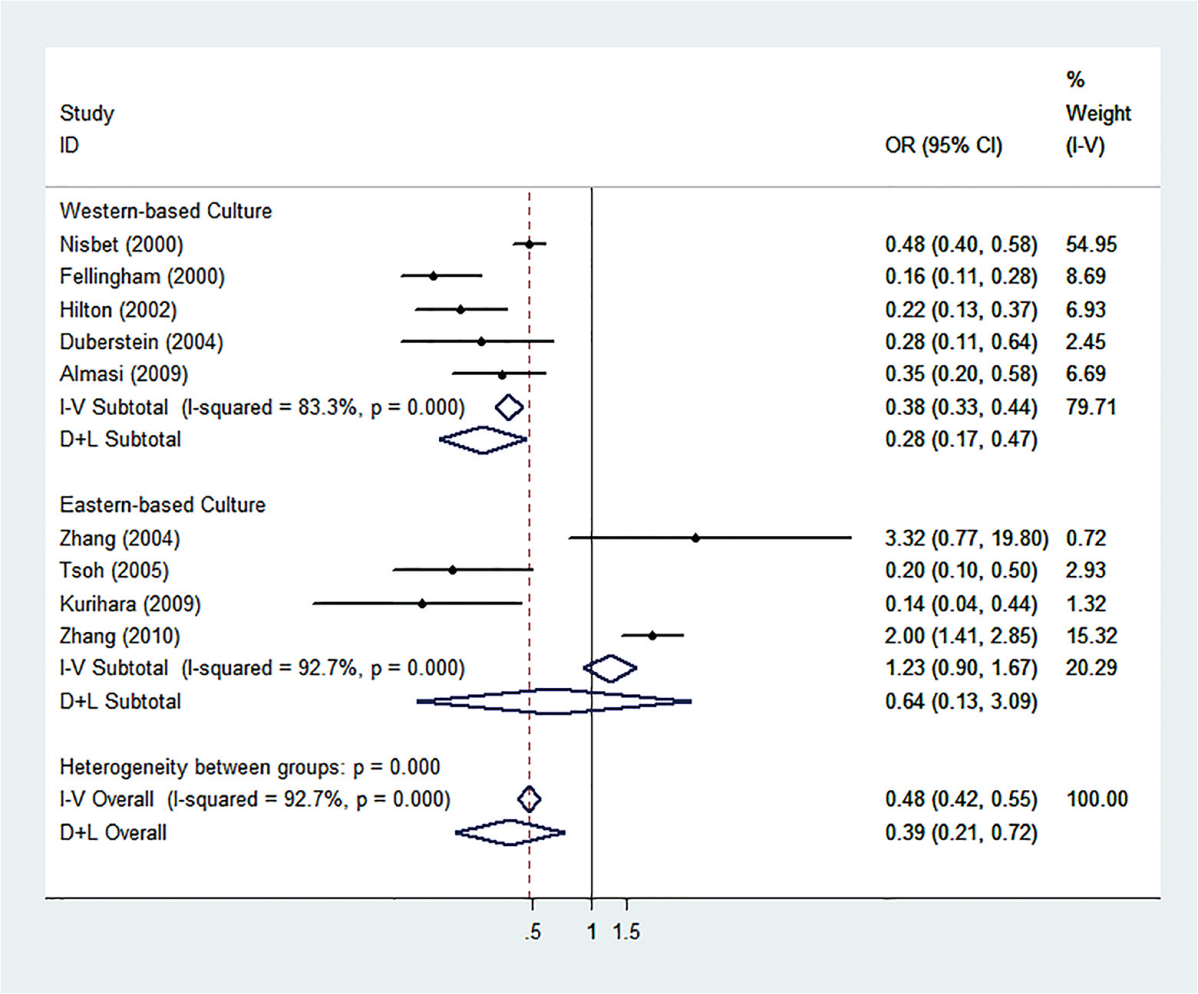
Sub-analysis of Eastern and Western-based Culture Settings. Forest plot of sub-analysis according to whether the study population resided in an eastern or western-based culture.

**Fig 3:**
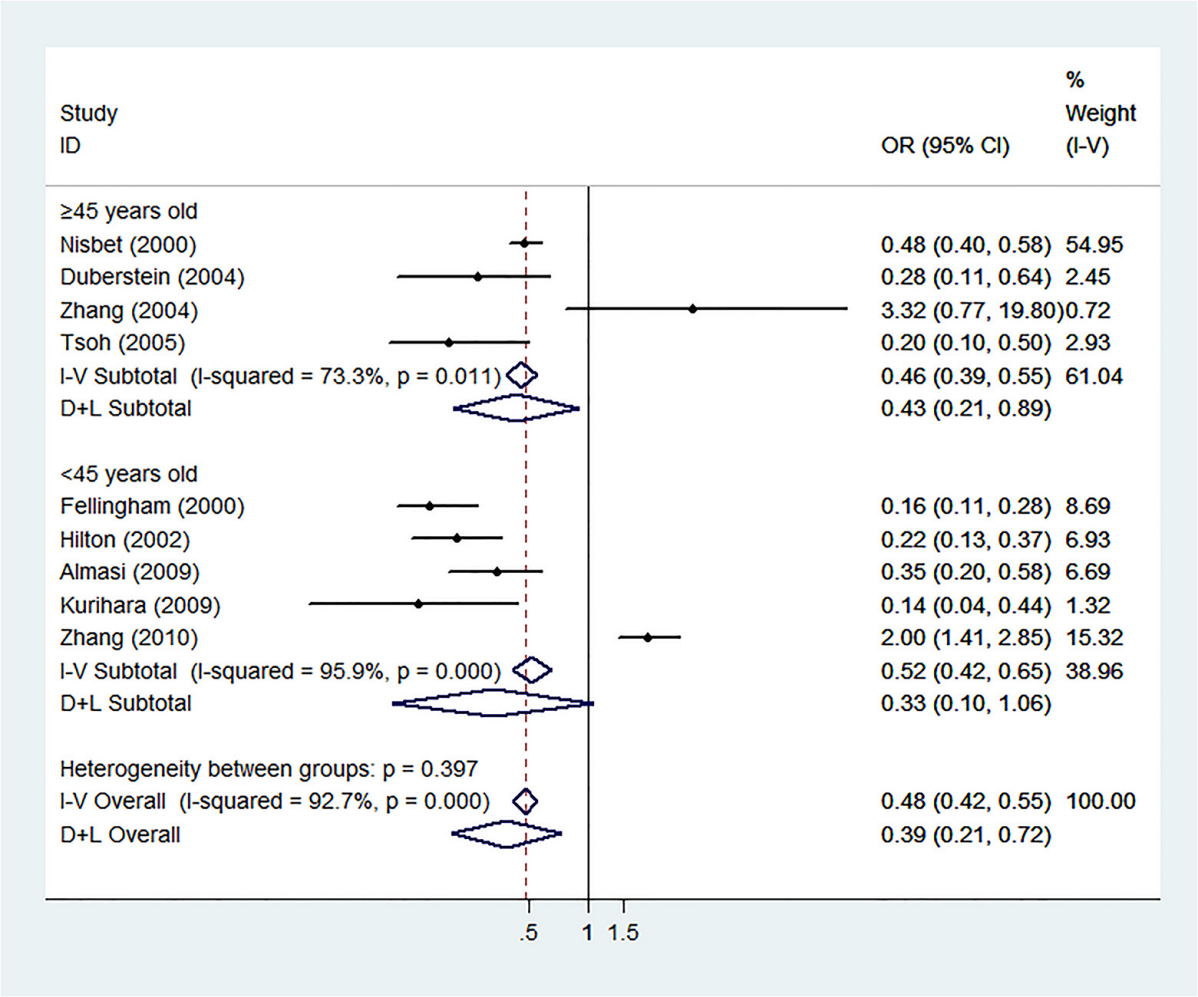
Sub-analysis of Younger and Older Age Groups. Forest plot of sub-analysis according to age group, with younger populations defined as less than 45 years of age and older groups as 45 years or older.

**Fig 4:**
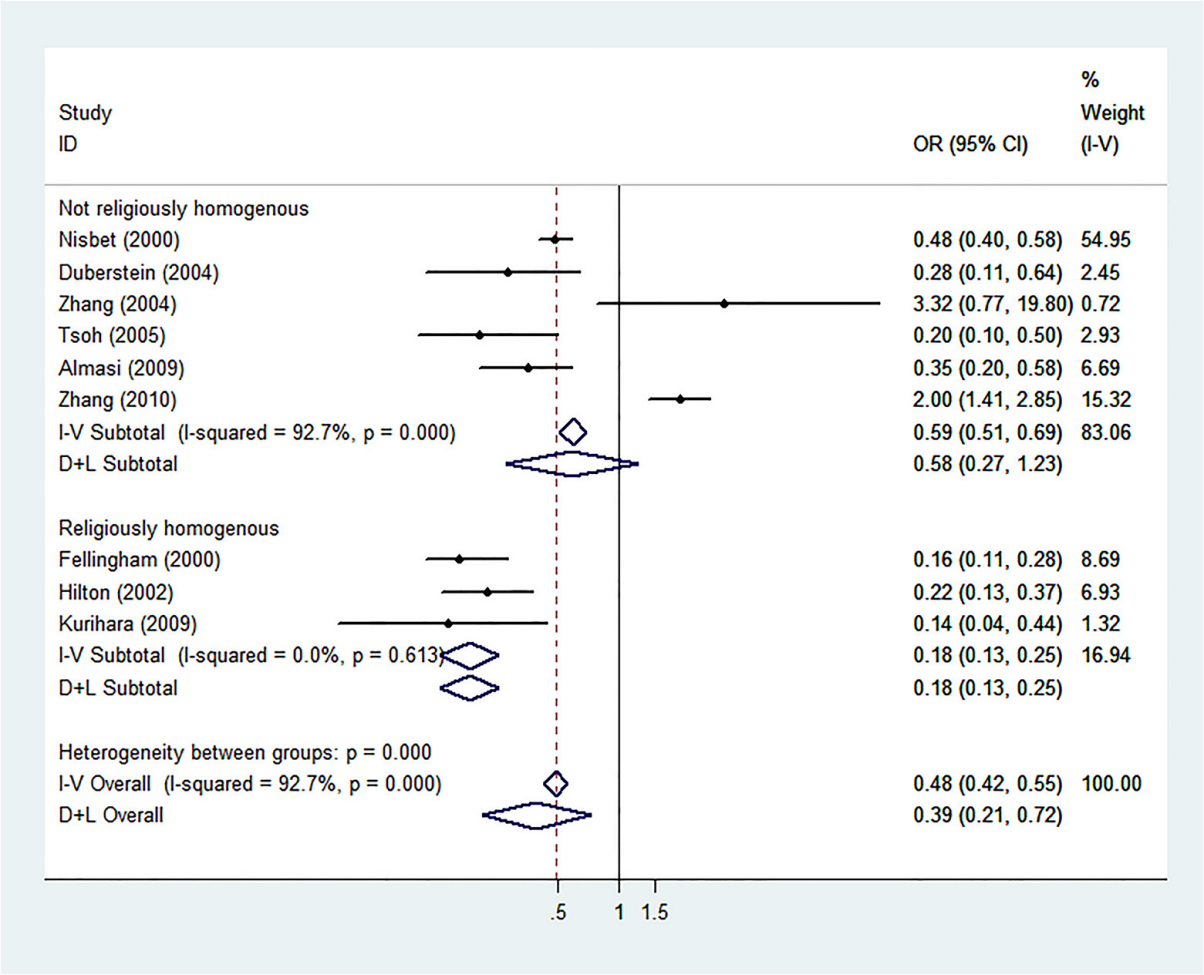
Sub-analysis of Religious Homogeneity or Heterogeneity. Forest plot for sub-analysis according to mixed or homogeneity of religion within the study population.

Sub-group analyses of western settings revealed significant results (REM: OR = 0.29, 95% CI: 0.18–0.46, p<0.001, I^2^ = 80%) but not in the analysis of eastern settings (REM: OR = 0.63, 95% CI: 0.13–3.12, p = 0.57, I^2^ = 92%). Analysis of the studies with younger study groups revealed no significant results (REM: OR = 0.33, 95% CI: 0.11–1.04, p = 0.058, I^2^ = 96%), but did so with older study groups (REM: OR = 0.42, 95% CI: 0.21–0.84, p = 0.01, I^2^ = 68%). Analysis of studies conducted in religiously homogeneous settings revealed significant results (REM: OR = 0.18, 95% CI: 0.13–0.26, p<0.001, I^2^ = 58.1%), but studies without this trait did not reach statistical significance (REM: OR = 0.57, 95% CI: 0.27–1.21, p = 0.14, I^2^ = 92%). Detailed results of these sub-analyses are shown in Figs 2, 3, and 4. The meta-regression for religious homogeneity was significant (p = 0.045), but non-significant for the other sub-analyses. The meta-regression to explore the effect of retrospective study designs versus prospective ones on the results also resulted in a non-significant relationship between study design and the meta-analysis outcome.

### Bias and Heterogeneity

**Fig 5:**
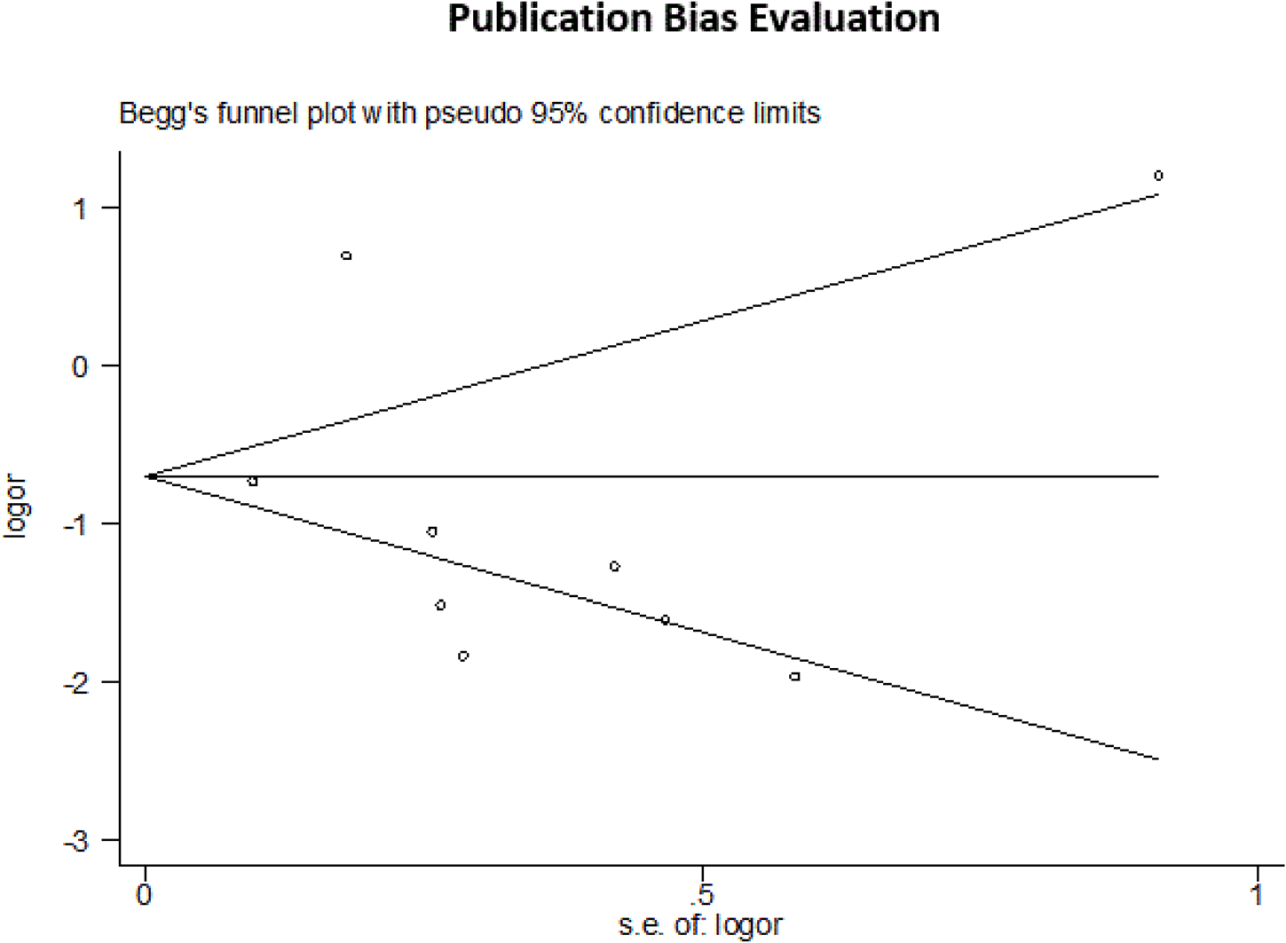
Publication Bias Evaluation. Funnel plot evaluating for publication bias among all nine studies included in the meta-analysis.

**Fig 6:**
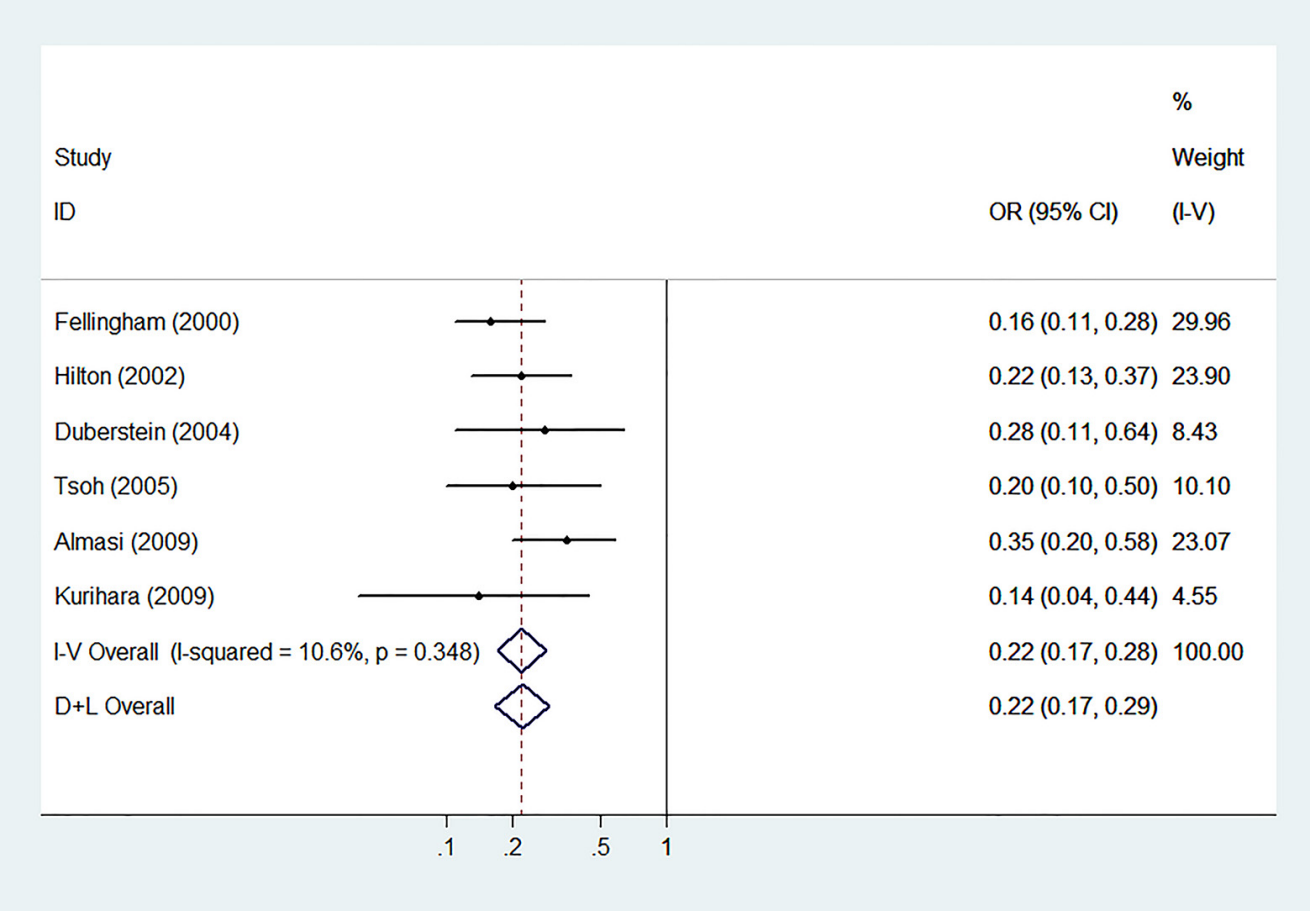
Meta-analysis With Minimal Heterogeneity. Forest plot of meta-analysis of remaining six articles after three articles were removed due to a significantly greater contribution of heterogeneity.

**Fig 7:**
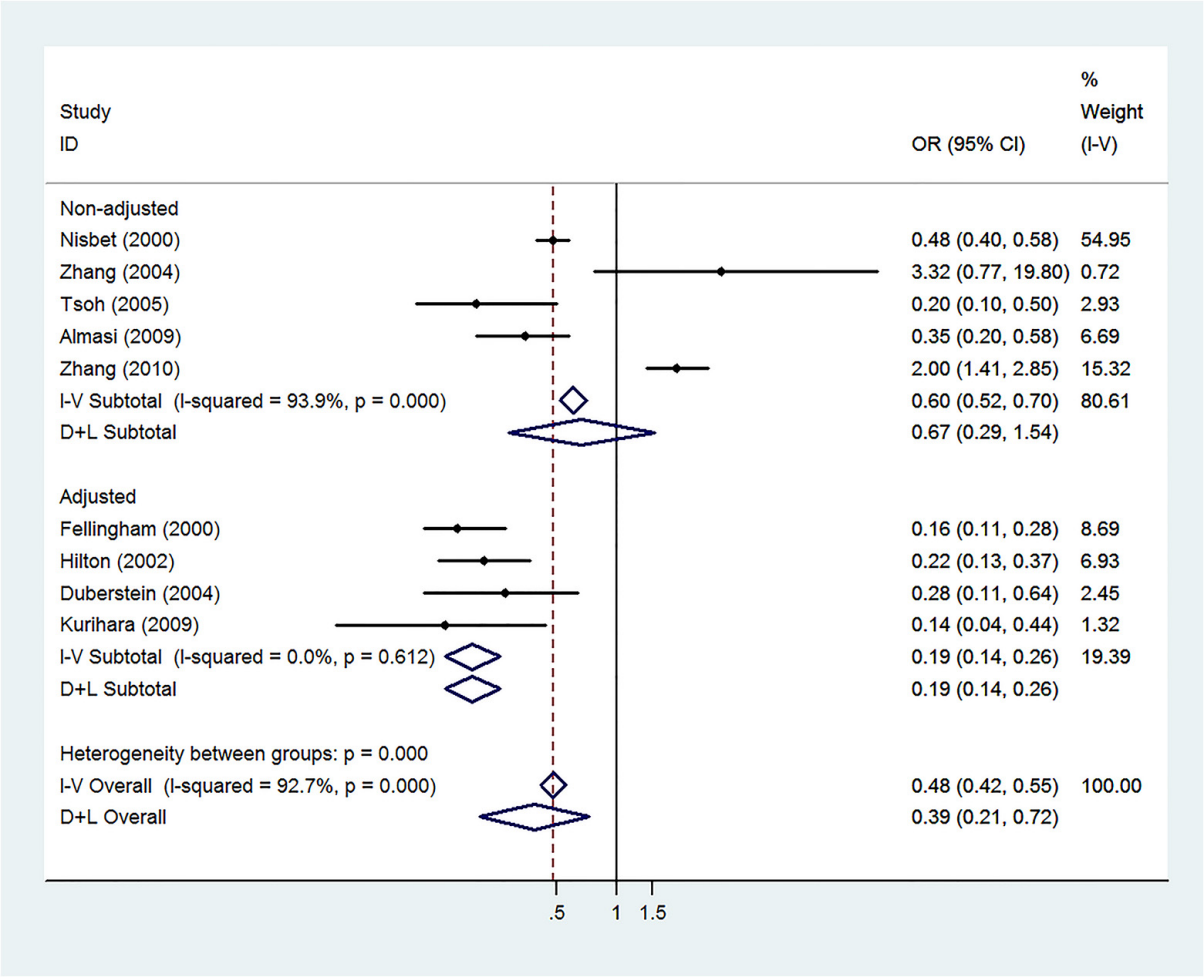
Sensitivity Analysis of Unadjusted and Adjusted Risk Estimates. Forest plot of meta-analysis of articles with adjusted risk estimates and those with unadjusted (calculated) risk estimates.

In the influence analysis, one article on the epidemiology of suicide among young people in rural China [23] significantly affected the pooled OR’s. Analyses for publication bias revealed none by the Egger’s test and Begg’s funnel plot (t = -0.94, p = 0.377 for both). The funnel plot is shown in Fig 5. Heterogeneity was high with Q = 101.24 with 8 degrees of freedom which is equivalent to an I^2^ of approximately 92% (p<0.001). After performing sequential omission with ‘hetred’, three articles [18, 23, 24] were found to significantly contribute heterogeneity. Removal of these three articles produced an I^2^ of 10.6% (p = 0.35). The forest plot after removal of these three articles is shown in Fig 6. The sensitivity analysis revealed high heterogeneity among the unadjusted estimates (I^2^ of 93.9%) and low heterogeneity among the adjusted estimates (I^2^ of 0.0%) which is shown in Fig 7.

## Discussion

In our meta-analysis, an overall significant protective relationship was found between religiosity and suicide completion. Upon exploration of certain sub-groups, this same relationship was found to be significant among studies that took place in western cultures, that studied an older population, and that was conducted in an area that was religiously homogenous.

In part, our results were unsurprising since many previous studies and reviews have documented religion to be protective against related ailments such as depression [39] and beneficial to health outcomes in general [5, 12, 40]. The possible mechanisms are numerous and include religious coping mechanisms during times of stress [41–44], social supports through religious communities [45, 46], or even specific teachings and behaviors for a given religion such as altruism and charity or abstaining from excessive alcohol consumption [43, 47]. What this meta-analysis uniquely adds however is observations across different contexts through the sub-analyses.

The fact that religion was a protective factor in western settings but not necessarily so in eastern settings was of particular interest. A cross-cultural case control study conducted in 2010 similarly found that religiosity was particularly strong in Brazil and South Africa but produced insignificant results in India and Vietnam [14]. People groups of the cultural east and west have vastly different views on the end of life. For example, eastern cultures have historically viewed suicide as an act of nobility and selflessness whereas western values have typically associated it with shame and cowardice, both views of which are linked with the dominant religious and spiritual beliefs of these areas [48]. This data supports that accounting for cultural values is vital to the success of suicide prevention strategy implementation in different parts of the world. For example, according to the World Values Survey in 2000, only 3% of participants in China considered religion as important [49]. However, more recently western religious beliefs have begun to surge in China. Research at Purdue University has estimated that the number of professing Christians in China has increased from about 7 million in 1990 to over 60 million in 2003 [50, 51]. He also predicts that by 2030, China may host the largest Christian population in the world [51]. This changing religious atmosphere and potential clash of values may also partially explain why religion may be a risk factor specifically in China [52]. Taken together, it therefore behooves the public health community to also take into consideration the changing face of religion in different countries.

That religion was shown to be a protective factor against suicide among the studies with older populations but not so with the younger population was somewhat puzzling. To our knowledge, no studies have been designed to intentionally determine if religion has a different impact on older versus younger populations regarding suicide. From a sociological perspective, it is possible that with older populations who may have lost community due to leaving their careers or children leaving the homes, religion then becomes a substantially larger part of their identity formation. Another possibility is that as health naturally deteriorates in old age, relying on religious coping behaviors may increase in the elderly. These are all consistent with the “lost theory” which suggests that the process of life naturally incurs continual loss of health, social position, relatives, friends, and life purpose [53]. This has special applicability for settings where the elderly make up a substantial proportion of the population such as in China [54].

Our last sub-group analysis was of particular interest both because the sub-analysis revealed a discrepancy between religiously homogenous settings and those that were not and because the meta-regression was also significant. This pattern has also been suggested by other studies performed in the U.S. [55, 56]. For example, a factor analysis of data from 1980 also found that religious homogeneity across the U.S. was inversely related to suicide rates [56]. In fact, its effect was estimated to be larger than and independent of other known religious factors such as church attendance. One may theorize that this may be due to a stronger sense of community derived from the principally identical belief structure that the majority of the population shares. This meta-analysis therefore also adds that this effect may be applied to other countries, and perhaps other religions, as well. This finding also supports the notion that suicide prevention strategies should be tailored to the particular social circumstances of a given people group.

The heterogeneity of this study was very high, the cause of which was isolated to three studies. Each of these three studies had unique characteristics that may explain their heterogeneity. One study was the only case-control design that did not use the psychological autopsy method, but instead used records of natural deaths as controls to compare against completed suicide cases [18]. The two other studies were conducted in rural areas, whereas all of the other studies occurred in cities or relatively urban areas [23, 24]. Belonging to an underserved community may imply different sets of risk factors for suicide than developed countries [15]. These three studies were also among those with unadjusted risk estimates, which likely explains the high heterogeneity that appeared in the sensitivity analysis. Lastly, the heterogeneity brought on by these three studies may also explain the asymmetry of the Begg’s funnel plot. The plot’s appearance may suggest publication bias, but after exclusion of these three studies, the funnel plot regained symmetry, suggesting that heterogeneity may be the primary contributor.

This meta-analysis has some limitations and considerations that should be considered when interpreting the results. First, we only selected studies whose outcomes were completed suicide. Studies that focused on other related outcomes, such as previous suicide attempt or depression, were not included since we were only interested the final stage of this disease process. However, these other elements are known risk factors for suicide completion. Therefore, future studies may broaden the selection criteria to further explore the effect of religion on the overall suicide burden. Second, the number of articles was relatively small, which may have limited the generalizability of our suggestions from countries that were not included. Future studies may build upon these results by possible expanding or reducing the number of search criteria. Third, the studies included in this analysis did not intentionally investigate the effect of specific types of religions on suicide risk. Though we reported a sub-analysis on religions in eastern and western cultures, each culture may include a variety of religious beliefs that may not otherwise be categorized together. For example, the studies performed in China captured data from individuals following Taoism, Christianity, Islam, and other belief systems which all widely differ from each other. Therefore, this meta-analysis does not suggest any relationships between specific religions and completed suicide. Fourth, it is altogether possible that religious beliefs may be abandoned well or shortly before committing suicide with no necessary causal relationship between these two events. Of course, such an occurrence is difficult to ascertain but must certainly be considered as a possible event that would affect how these results are interpreted. Lastly, the time period eligibility for collecting data was limited. Therefore, future meta-analyses that focus on this topic may need to search for relevant literature before the year 2000 to capture any effects that may inform the relationship between religion and suicide today.

In conclusion, this study’s results further support the notion that religion plays an overall protective role against completed suicide. Additionally, our sub-analyses have also clarified that this protective relationship varies depending on the individual and the society at large. This data can be used to aid the design of suicide prevention policies, programs, and strategies in a variety of settings in order to target the people groups at highest risk.

## Supporting Information

S1 Checklist PRISMA Checklist. (DOC)

S1 Fig Influence Analysis. (TIF)

S2 Fig Begg’s Funnel Plot with Minimal Heterogeneity. (TIF)

S1 Table Full Dataset. This table includes the data we used to conduct the analyses in this study. Cases were defined as those who had committed suicide. Controls were defined as those who were either living comparisons or had died natural deaths. Studies by Fellingham, et. al and Hilton, et. al were retrospective and all participants had committed suicide. Therefore, these studies’ controls were the non-religious cases. A 1 for “Adjusted OR/CI” indicates that the adjusted values were included in the study, whereas a 0 indicates that the OR’s and 95% CI’s were calculated by the study investigators based off of the exposed and non-exposed cases and controls.
(DOCX)

S2 Table Literature Quality Assessment. This study used the Newcastle-Ottawa Scale (NOS) for Assessing the Quality of Nonrandomized Studies in Meta-Analysis. Entries with the * symbol represent earning one star with the total number of stars in the right-most column.
(DOCX)
