# Supplementary figures and images for: Religion and Completed Suicide: a Meta-Analysis
Source: PLoS One. 2015 Jun 25;10(6):e0131715. doi: 10.1371/journal.pone.0131715 (PMC4482518; doi:10.1371/journal.pone.0131715)

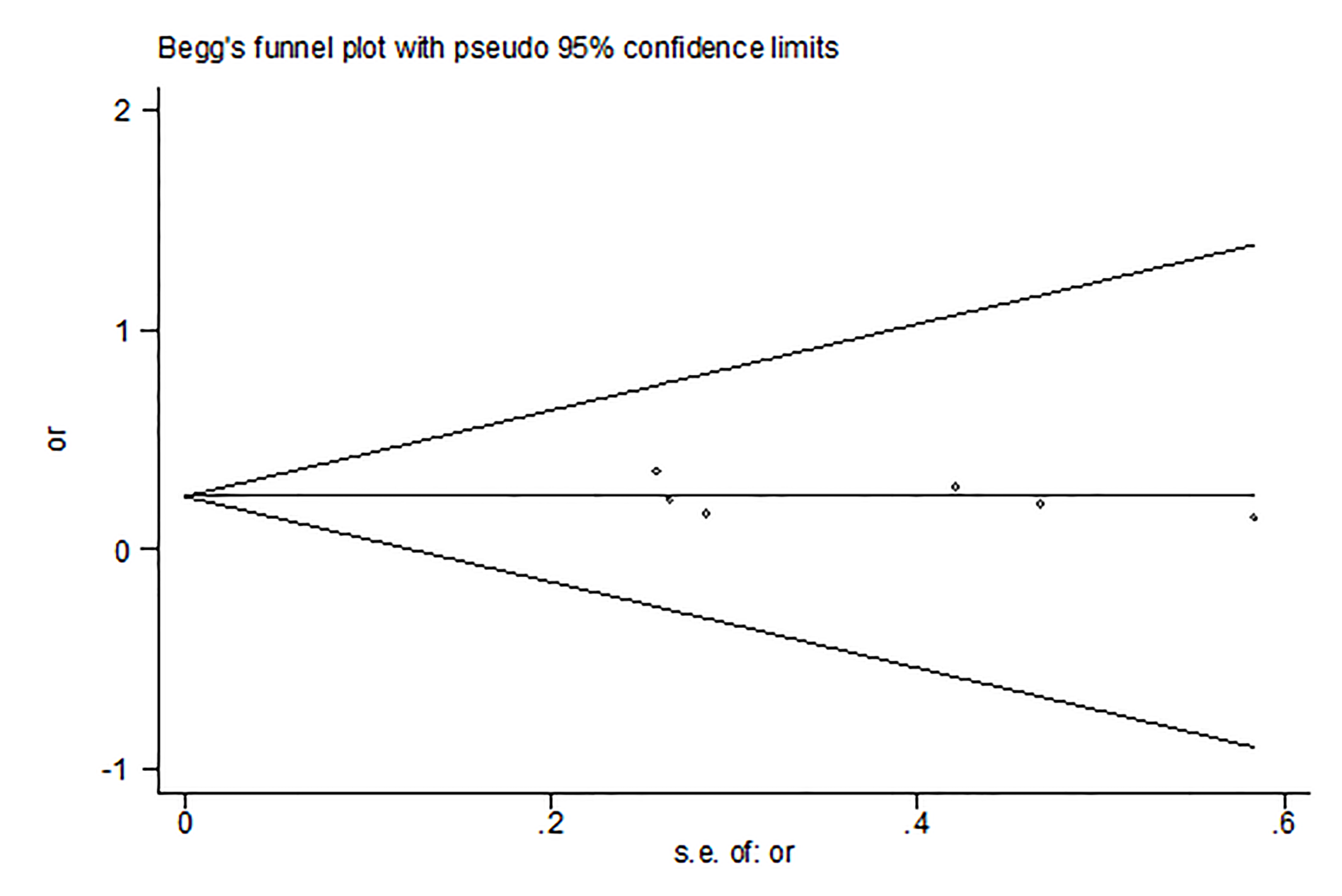

Supplement: S2 Fig — (TIF) [file pone.0131715.s003.tif]
