# Supplementary material for: Religion and Completed Suicide: a Meta-Analysis
Source: PLoS One. 2015 Jun 25;10(6):e0131715. doi: 10.1371/journal.pone.0131715 (PMC4482518; doi:10.1371/journal.pone.0131715)
Supplement: S1 Table — This table includes the data we used to conduct the analyses in this study. Cases were defined as those who had committed suicide. Controls were defined as those who were either living comparisons or had died natural deaths. Studies by Fellingham, et. al and Hilton, et. al were retrospective and all participants had committed suicide. Therefore, these studies’ controls were the non-religious cases. A 1 for “Adjusted OR/CI” indicates that the adjusted values were included in the study, whereas a 0 indicates that the OR’s and 95% CI’s were calculated by the study investigators based off of the exposed and non-exposed cases and controls. (DOCX) [file pone.0131715.s004.docx]

| **First Author** | Nisbet | Fellingham | Hilton | Duberstein | Zhang | Tsoh | Almasi | Kurihara | Zhang |
| --- | --- | --- | --- | --- | --- | --- | --- | --- | --- |
| **Year** | 2000 | 2000 | 2002 | 2004 | 2004 | 2005 | 2009 | 2009 | 2010 |
| **Location** | USA | USA (Utah) | USA (Utah) | USA (NY) | China | China | Hungary | Bali | China |
| **Study Design** | Case-control | Retrospective cohort | Retrospective cohort | Case-control psychological autopsy | Case-control psychological autopsy | Case-control psychological autopsy | Case-control psychological autopsy | Case-control psychological autopsy | Case-control psychological autopsy |
| **Definition of Religion** | Church attendance (per week) | Priesthood (recorded) | Priesthood (recorded) | Practicing a religion | Claiming a religion, belief in God/afterlife | Religion considered salient | Practicing a religion | Practicing a religion (temple anniversaries) | Claiming a religion, belief in God/afterlife, church attendance |
| **Total cases** | 584 | 215 | 317 | 86 | 66 | 67 | 192 | 60 | 392 |
| **Religious cases** | 242 | 59 | 95 | 28 | 9 | 11 | 30 | 33 | 113 |
| **Non-religious cases** | 342 | 156 | 222 | 57 | 57 | 56 | 162 | 27 | 279 |
| **Total controls** | 4279 | 0 | 0 | 86 | 66 | 91 | 192 | 120 | 416 |
| **Religious controls** | 2541 | 0 | 0 | 50 | 3 | 44 | 67 | 114 | 70 |
| **Non-religious controls** | 1738 | 0 | 0 | 36 | 63 | 47 | 125 | 6 | 346 |
| **Total N** | 4863 | 215 | 317 | 158 | 180 | 388 | 172 | 132 | 808 |
| **OR** | 0.48 | 0.16 | 0.22 | 0.28 | 3.32 | 0.20 | 0.35 | 0.14 | 2 |
| **LCI** | 0.40 | 0.11 | 0.13 | 0.11 | 0.77 | 0.10 | 0.20 | 0.04 | 1.41 |
| **UCI** | 0.58 | 0.28 | 0.37 | 0.64 | 19.8 | 0.5 | 0.58 | 0.44 | 2.85 |
| **Young** | 0 | 1 | 1 | 0 | 0 | 0 | 1 | 1 | 1 |
| **Religiously Homogenous** | 0 | 1 | 1 | 0 | 0 | 0 | 0 | 1 | 0 |
| **Western culture** | 1 | 1 | 1 | 1 | 0 | 0 | 1 | 0 | 0 |
| **Adjusted OR/CI** | 0 | 1 | 1 | 1 | 0 | 0 | 0 | 1 | 0 |
| **Retrospective study** | 0 | 1 | 1 | 0 | 0 | 0 | 0 | 0 | 0 |
| **Mean age** | 71.25 | . | . | 67.5 | 45 | 73 | 43 | 41.4 | 26 |
