# Supplementary material for: Religion and Completed Suicide: a Meta-Analysis
Source: PLoS One. 2015 Jun 25;10(6):e0131715. doi: 10.1371/journal.pone.0131715 (PMC4482518; doi:10.1371/journal.pone.0131715)
Supplement: S2 Table — This study used the Newcastle-Ottawa Scale (NOS) for Assessing the Quality of Nonrandomized Studies in Meta-Analysis. Entries with the * symbol represent earning one star with the total number of stars in the right-most column. (DOCX) [file pone.0131715.s005.docx]

| **First Author** | Almasi | Tsoh | Zhang | Duberstein | Nisbet | Kurihara | Fellingham | Hilton | Zhang |
| --- | --- | --- | --- | --- | --- | --- | --- | --- | --- |
| **Year** | 2009 | 2005 | 2010 | 2004 | 2000 | 2009 | 2000 | 2002 | 2004 |
| **Study Design** | Case-control psychological autopsy | Case-control psychological autopsy | Case-control psychological autopsy | Case-control psychological autopsy | Case-control | Case-control psychological autopsy | Retrospective cohort | Retrospective cohort | Case-control psychological autopsy |
| **Defining cases (i.e. suicide)** | record link | record link | record link | independently measured* | record link | independently measured* | truly representative* | truly representative* | independently measured* |
| **Cases appropriately selected** | well-selected* | well-selected* | well-selected* | well-selected* | well-selected* | well-selected* | selected from same source as controls* | selected from same source as controls* | well-selected* |
| **Source of controls** | Community* | community* | community* | community* | community* | community* | records* | records* | community* |
| **Defining controls** | no h/o suicide* | no h/o suicide* | no h/o suicide* | no h/o suicide* | no h/o suicide* | no h/o suicide* | suicide not present earlier* | suicide not present earlier* | no h/o suicide* |
| **Controlled for religion** | Yes* | yes* | yes* | yes* | yes* | yes* | yes* | yes* | yes* |
| **Controlled for social interaction** | Yes* | yes* | yes* | yes* | yes* | yes* | no | no | yes* |
| **Assessment of outcome/exposure** | interview not blinded to case/control status | interview not blinded to case/control status | interview not blinded to case/control status | interview not blinded to case/control status | interview not blinded to case/control status | interview not blinded to case/control status | record link* | record link* | interview not blinded to case/control status |
| **Method for ascertaining cases/controls the same** | no | no | no | no | Yes* | Does not mention | Adequate follow-up* | Adequate follow-up* | Yes* |
| **Non-response rate within 10%** | no | no | Does not mention | no | Yes* | Yes* | Does not mention | Does not mention | Yes* |
| **Total Stars** | 5 | 5 | 5 | 6 | 7 | 7 | 7 | 7 | 8 |
